# First-Principles Calculation and Experimental Study on Interface Stability, Electronic Characteristics, and Mechanical Properties of WC-Co-Y Cemented Carbide

**DOI:** 10.3390/ma19020441

**Published:** 2026-01-22

**Authors:** Zewen Li, Hao Chen, Liyong Chen, Jianbo Zhang, Fan Zhang, Xiaolong Xie

**Affiliations:** 1 School of Materials Science and Engineering, Jiangxi University of Science and Technology, Ganzhou 341000, China; 15797643385@163.com (Z.L.); cly@jxust.edu.cn (L.C.); zhang4318@163.com (J.Z.); ijwqwz4132@163.com (F.Z.); xiexiaolong@jxust.edu.cn (X.X.); 2 School of Education, Nanchang Institute of Science and Technology, Nanchang 330108, China; 3 Engineering Research of Center of High-Efficiency Development and Application Technology of Tungsten Resources, Ministry of Education, Jiangxi University of Science and Technology, Ganzhou 341000, China; 4 Collaborative Innovation Center for Development and Utilization of Rare Metal Resources Co-Sponsored by Ministry of Education and Jiangxi Province, Jiangxi University of Science and Technology, Ganzhou 341000, China

**Keywords:** first-principles calculation, interface model, electronic characteristics, hardness, wear rate

## Abstract

**Highlights:**

**What are the main findings?**

- Y doping reduces WC/Co interfacial energy and enhances bonding strength. Y-W orbital hybridization forms strong covalent bonds at the atomic interface.
- Material achieves dual improvement: Hardness (1454 HV) and toughness (9.84 MPa·m^1^/^2^).
- Optimal 0.5 wt.% Y refines grains and uniformly distributes the Co binder phase. Wear resistance is significantly improved, with the lowest wear rate at 0.5 wt.% Y.

**What are the implications of the main findings?**

- Provides an atomic-scale mechanism for rare earth-enhanced cemented carbides and offers a design strategy for co-optimizing hardness and toughness in composites;
- Validates the integration of first-principles calculations with experimental validation;
- Identifies an optimal Y doping threshold for superior comprehensive properties.

**Abstract:**

This study aims to clarify the optimization mechanism of yttrium (Y) doping on the interfacial bonding and macroscopic properties of WC/Co cemented carbides, with the goal of achieving materials that combine high hardness, high toughness, and excellent wear resistance through interfacial regulation. Combining first-principles calculations and experimental verification, the interfacial energy, density of states, and charge density of WC/Co and WC/CoY interfaces were systematically investigated. Three alloys (WC-10Co, WC-10Co-0.5Y, and WC-10Co-1Y) were prepared, and the effects of Y addition were quantitatively evaluated through microstructural characterization, mechanical testing, and tribological experiments. The calculation results indicate that Y doping reduces interfacial energy, enhances interfacial bonding, and increases surface energy, which contributes to improved toughness. At the atomic scale, the orbital hybridization between Y and W promotes the formation of strong covalent bonds at the interface, thereby enhancing interfacial bonding strength. The experimental results show that the introduction of Y significantly improves the overall performance of the material, with the alloy containing 0.5 wt.% Y exhibiting the best performance. Its Vickers hardness reaches (1454 ± 1.3) HV, fracture toughness is (9.84 ± 0.15) MPa·m^1/2^, and the wear rate is as low as 0.794 × 10^−5^ mm^3^·N^−1^·m^−1^.

## 1. Introduction

WC-based cemented carbide is a metal–ceramic composite consisting of a hard, wear-resistant tungsten carbide skeleton bound by a ductile cobalt metal binder [1,2,3]. This material integrates an exceptional combination of ultra-high hardness, outstanding wear resistance, good toughness, high strength, and excellent hot hardness, making it the preferred choice for cutting tools, wear-resistant components, and other demanding service conditions [4,5,6,7]. As the applications for cemented carbides continue to expand, performance requirements are constantly increasing. Consequently, developing new grades of cemented carbide with enhanced overall properties has become a key focus of current research.

The hardness and toughness of cemented carbide exhibit an inverse correlation. Enhancing hardness typically results in reduced toughness, which poses challenges in meeting the demands for high load-bearing capacity and fatigue resistance under extreme operating conditions [8]. Additionally, the cobalt (Co) binder phase is susceptible to softening and oxidation at elevated temperatures, leading to a notable decline in the alloy’s wear resistance and strength. Furthermore, abnormal grain growth of WC during sintering can degrade material performance, and conventional grain inhibitors (e.g., Cr, V, Mo) often provide limited effectiveness [9,10,11]. As a result, traditional cemented carbide products are increasingly unable to support advances in emerging technology sectors and are gradually falling short of current market requirements. To overcome these limitations, elemental doping has emerged as a key strategy for optimizing WC–Co cemented carbides. Rare earth elements (REEs) such as La, Ce, and Y—often referred to as “industrial vitamins”—possess unique electronic structures and high chemical activity, enabling them to refine grains, purify interfaces, and improve oxidation resistance [12,13,14,15,16]. In recent years, researchers have actively investigated the influence of REEs on cemented carbide properties [17,18,19]. For instance, Xia et al. [20] used conventional powder metallurgy to study the effects of La_2_O_3_ and Y_2_O_3_ on the microstructure and mechanical properties of recycled WC–12Co–2Ni carbide. They found that appropriate additions of rare earth oxides significantly enhanced the alloy’s toughness and flexural strength. Similarly, Lu et al. [21] reported that adding 0.25 wt% Y_2_O_3_ and 0.25 wt% VC via low-pressure sintering markedly improved the microstructure and overall mechanical properties of WC–10Co cemented carbide. In another study, Yang et al. [22] employed pressure-assisted sintering to examine the impact of Y_2_O_3_ powder on WC–8Co carbide. Their results demonstrated that rare earth elements notably enhanced mechanical properties and wear resistance by pinning grain boundaries, strengthening intra-grain structure, and refining the grains. While experiments have confirmed that REEs can enhance the performance of cemented carbides [23,24,25,26], their doping mechanisms at the microscopic level remain difficult to elucidate through experimental methods alone. First-principles calculations, however, offer an effective approach to uncovering these mechanisms [27,28,29]. Among various computational models, the interface model is particularly suitable for studying atomic doping in polycrystalline materials and has been widely applied to analyze diverse interface systems, including metal/metal and metal/ceramic interfaces [30,31,32]. For example, Hao et al. [33] employed first-principles calculations to construct interface models and computed the elastic constants, electron distribution characteristics, and thermodynamic properties of WC/Co with and without Y doping. Their work predicted an overall improvement in the performance of the doped alloy. Likewise, Zhang et al. [34] used first-principles calculations to explore how rare earth elements affect the interfacial bonding in WC/α-Fe cemented carbide. They found that doping with Sc and Pr enhanced interfacial strength, whereas Nb strengthened the interface regardless of its doping site.

The advent of first-principles calculations has provided an atomic-scale perspective for elucidating doping mechanisms. By constructing WC/Co interface models and computing key properties such as interfacial energy, electronic structure, and diffusion kinetics, the role of dopant elements and their effects on material performance can be predicted with high reliability, thereby offering valuable guidance for experimental design [35,36,37]. Although previous studies have investigated the effects of rare earth elements on cemented carbides, most remain confined to either experimental observations or isolated theoretical predictions. There is still a lack of systematic work that closely integrates first-principles calculations with experimental validation to elucidate the atomic-scale mechanisms behind performance enhancement. To address this gap, the present study employs a combined computational-experimental methodology to investigate Y-doped WC–Co cemented carbide. We aim not only to predict the interfacial and electronic properties through first-principles modeling but also to experimentally verify these predictions through microstructure characterization and mechanical testing. This dual approach allows us to uncover the underlying strengthening mechanisms of Y doping from the atomic to the macroscopic level, providing a comprehensive understanding that is often missing in conventional studies. Ultimately, this work seeks to develop a new grade of cemented carbide that exhibits high hardness, high toughness, and superior wear resistance.

## 2. Materials and Methods

### 2.1. Models and Methods

**Figure 1:**
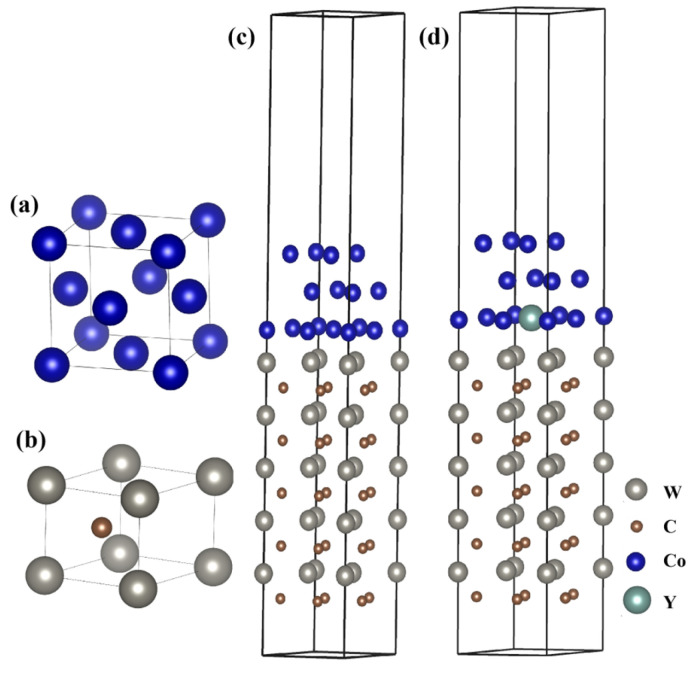
Interface models: (**a**) Co; (**b**)WC; (**c**) WC/Co; (**d**) WC/CoY.

This paper mainly takes WC/Co and WC/Co-doped rare earth element Y as the research objects, and mainly studies the interface energy and electronic properties of the models before and after doping. Co, as a binder, shows excellent wettability to the surface of WC particles during the liquid-phase sintering process. Molten Co fills the gaps between WC particles through capillary action [38]. This enables the Co phase to penetrate between the WC and WC grain boundaries, forming the WC/Co/WC phase boundary. The selection of crystallographic orientations for the interface model is crucial for accurately representing the dominant interfacial configuration in real polycrystalline cemented carbides. Previous studies, along with our own surface energy calculations, have consistently shown that the WC(0001) surface is the most stable low-energy surface in WC crystals and is commonly adopted as the representative surface for interface modeling with the Co binder [33,39]. Similarly, the close-packed Co(111) plane possesses the lowest surface energy in the Co crystal structure. More importantly, the WC(0001) and Co(111) planes exhibit a favorable crystallographic match, which minimizes the interfacial misfit strain. To construct a coherent or semi-coherent interface slab model for first-principles calculations, we aligned the in-plane lattice vectors of WC(0001) and Co(111). The lattice parameters were taken from fully relaxed bulk WC (a = 2.906 Å, c = 2.837 Å) and FCC-Co (a = 3.544 Å) structures. A supercell approach was employed to accommodate the lattice mismatch. The final interfacial model was built with a manageable supercell size where the in-plane lattice mismatch was reduced to below 5%, ensuring computational feasibility while maintaining physical relevance. In this model, the Co slab was subjected to a slight biaxial strain to coherently match the WC substrate, as the WC phase is significantly harder and its lattice is less prone to distortion during sintering. The resulting interface model, representing a stable, low-energy configuration, is shown in Figure 1. The crystal structures of WC and Co are shown in Figure 1a,b. The adsorption of Co on the WC surface, the segregation of grain boundaries, and the displacement of phase boundaries are shown in Figure 1c. The research indicates that in the microstructure of rare earth cemented carbide, rare earth elements exist in the grain boundaries and the Co-bonded phase, but are not detected in the WC phase [40]. In this paper, through the energy calculation of the four doping positions at the interface, it is found that the Y-doped structure at the position shown in Figure 1d has the lowest and most stable energy. Therefore, the WC/CoY interface model, as shown in Figure 1d, is established.

All DFT calculations in this study were performed using the VASP. The PAW method was employed to describe the electron-ion interactions. The exchange-correlation potential was selected as the PBE functional within the GGA. The following key parameters and convergence criteria were strictly applied in the calculations: The plane-wave cutoff energy was set to 450 eV, determined through convergence tests on the total energies of bulk WC and Co, ensuring that further increase in the cutoff energy changed the energy by less than 1 meV per atom; for the interface slab model, a 7 × 7 × 1 k-point mesh was employed for Brillouin-zone integration. The interface model consists of 10 atomic layers of WC and 3 layers of Co (or CoY), with a 15 Å vacuum layer introduced perpendicular to the interface to eliminate spurious interactions between periodic images. To mimic a semi-infinite substrate, the bottom six layers of WC atoms were fixed at their bulk positions, whereas all atoms in the Co (or CoY) layers, as well as the top layers of WC, were fully relaxed. The convergence threshold for the total energy change in the electron self-consistent field (SCF) cycle was set to 1 × 10^−5^ eV. During relaxation, a Gaussian smearing of 0.05 eV was applied to improve Brillouin-zone integration convergence, while static calculations for density of states and charge density analysis used the tetrahedron method with Blöchl corrections. Geometry optimization of ionic relaxation was performed until the Hellmann–Feynman forces on all free atoms fell below 0.01 eV/Å.

### 2.2. Experimental Process

**Figure 2:**
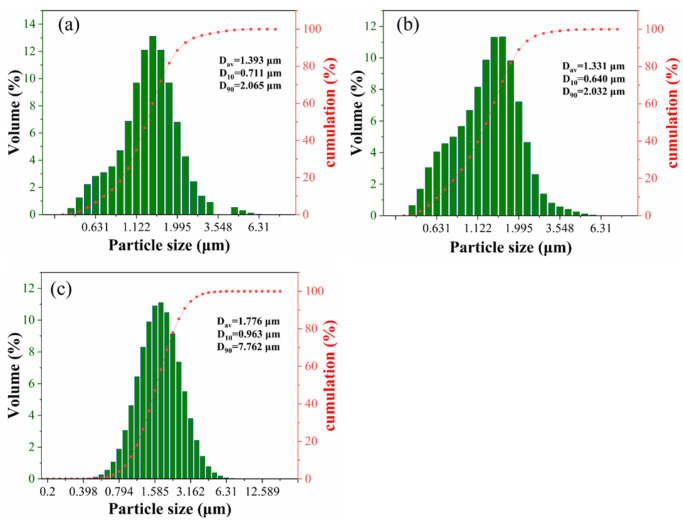
Particle size: (**a**) WC; (**b**) Co; (**c**) Y2O3.

**Figure 3:**
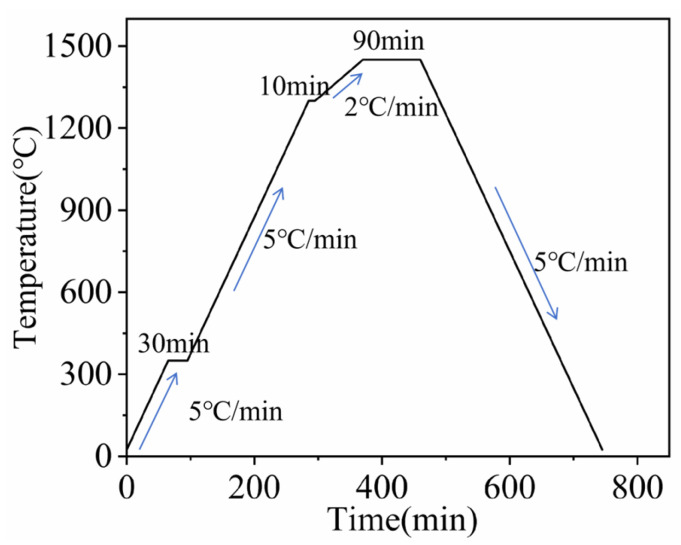
Sintering process curve.

Ball milling was carried out using Co powder (Provided by Hebei Hangbai Metal Materials Co., Ltd., Tangshan, China, Purity > 99.9%, 1.0–3.0 μm, 10 wt.%), rare earth powder (Provided by Aladdin Holdings Group Co., Ltd., Shanghai, China, Purity > 99.99%, 1.0–3.0 μm, 0.5 wt.% or 1 wt.% of Y_2_O_3_), WC powder (Provided by Hebei Hangbai Metal Materials Co., Ltd. Purity > 99.99%, 1.0–3.0 μm, surplus), and liquid paraffin containing 2.0% as raw materials. The particle size distributions of three powder samples were determined using a laser particle size analyzer (Model: winner2005), Jinan Nanoparticle Instrument Co., Ltd., Jinan, China, as shown in Figure 2. The experiment adopted hard alloy ball milling jars and hard alloy balls with diameters of 6, 8, and 10 mm, respectively, to avoid introducing impurities and contaminating the raw materials. The ball milling solvent is anhydrous ethanol, the ratio of balls to materials is 8:1, the rotational speed is 220 r/min, and the ball milling time is 12 h. After ball milling, the mixed powder was vacuum dried at 80 ℃ for 6 h. WC-Co-Y composite powder was obtained after 80-mesh sieving. Take 15 g of the composite powder and load it into a hard alloy mold with a diameter of 20 mm. It is formed by holding the pressure at 300 MPa for 20 seconds using a hydraulic press, Taohong Intelligent Equipment Co., Ltd., Shenzhen, China. The green compact was sintered in an atmosphere sintering furnace (model GSL-1600X), Hefei Jinko Materials Technology Co., Ltd., Hefei, China. The sintering process curve is shown in Figure 3. The sintered samples were cut into 10 mm × 10 mm cubes with a thickness of 10 mm using a DK7740 wire cutting machine, Kunshan Ruiyun Mechanical Technology Co., Ltd., Kunshan, China, and then mounted using an XQ-2B metallographic mounting press, Guangjie Machinery Co., Ltd., Jinan, China. The samples were subjected to coarse and fine grinding with diamond grinding disks, and finally polished with diamond paste to obtain specimens for scanning electron microscopy, Beijing Zhongke Keyi Co., Ltd., Beijing, China. After obtaining the cemented carbide, the sintered samples were characterized by scanning electron microscopy (model MLA-650), Beijing Zhongke Keyi Co., Ltd., Beijing, China, combined with energy-dispersive X-ray spectroscopy (including point scanning and area scanning). Tests were also conducted for Vickers hardness (with a load of 30 kgf), fracture toughness (using the indentation method with a load of 30 kgf), and friction and wear properties (through a 1 h reciprocating friction and wear test).

The surface microstructure, friction, wear morphology, and elemental distribution of the samples were analyzed using a scanning electron microscope (MLA-650) equipped with an energy-dispersive spectrometer (EDS). Sample density was measured with an AU-600ME alloy densitometer, Hangzhou Jinmai Instrument Co., Ltd., Hangzhou, China. Hardness and fracture toughness were evaluated using a FUTURE TECH fully automatic micro-Vickers hardness testing system, Naboo Detection Technology Co., Ltd., Shanghai, China. Wear tests were performed on an HRS-2M reciprocating sliding wear tester, Lanzhou Zhongke KaihuaNaboo Detection Technology Co., Ltd., Lanzhou, China. The three-dimensional topography of the wear tracks was characterized with a NanoMap 500 LS 3D, Leichuang 3D Technology Co., Ltd., Guangzhou, China, surface profiler, while two-dimensional wear profiles were obtained using an MT-500, Xiamen Deyou Testing Instrument Co., Ltd., Xiamen, China, wear testing machine.

## 3. Results and Discussion

### 3.1. Interface Energy

The stability of the WC/Co interface and the Y-doped WC/CoY interface directly influences the mechanical properties of cemented carbide. To evaluate the interfacial bonding strength at the atomic scale, this study systematically calculates and compares the interfacial energy (γ) of the two interfaces based on first-principles calculations and the constructed interface model (Figure 1). Interfacial energy is a key parameter for evaluating the thermodynamic stability of heterogeneous interfaces: the lower its value, the more energetically stable the interface, the stronger the interatomic bonding, and the less prone it is to separation. Surface energy (E*_surf_*) is one of the important parameters for measuring this stability. Adhesion work (W*_ab_*) quantitatively characterizes the minimum work required to separate two contacting substances from the interface, directly reflecting the interfacial bonding strength. When surfaces a and surface b combine to form an interface, the atoms at the interface undergo rearrangement, causing a change in the system’s energy. The additional energy stored during this process is the interface energy (γ). The interface energy can be calculated through the following formula [41,42,43]:

(1) $$\begin{array}{l} E_{surf}=\frac{E_{slab}-\left(\frac{N_{slab}}{N_{bulk}}\right)E_{bulk}}{2A} \end{array}$$

(2) $$W_{ab}=\frac{E_{a}+E_{b}-E_{ab}}{S}$$

(3) $$\gamma =\sigma_{1}+\sigma_{2}-W_{ab}$$

Here, E*_slab_* represents the surface energy of the optimized unit cell; E*_bulk_* represents the total energy of the optimized unit cell. N*_slab_* represents the number of atoms on the crystal plane; N*_bulk_* represents the total number of atoms in the unit cell. A represents the surface area of the unit cell; E*_ab_* represents the total energy of the interface formed between surfaces *a* and *b*. E*_a_* represents the total energy on the a side. E*_b_* is the total energy of side *b*. S represents the interface area; σ_1_ and σ_2_ represent the surface energies of surface *a* and surface *b*, respectively.

The calculated interfacial energies for WC/Co and WC/CoY are presented in Table 1. Analysis indicates that Y doping significantly alters the interfacial properties: the interfacial formation energy (γ) of WC/CoY is lower than that of the undoped WC/Co interface, implying that the introduction of Y enhances interatomic interactions at the interface and improves interfacial bonding stability. Meanwhile, WC/CoY exhibits a higher surface energy, which theoretically means that more energy is required for fracture, contributing to improved macroscopic toughness of the material. In summary, the calculation results demonstrate that Y doping reduces interfacial energy and increases surface energy, thereby strengthening interfacial bonding and enhancing material toughness.

### 3.2. Electronic Density of States

**Figure 4:**
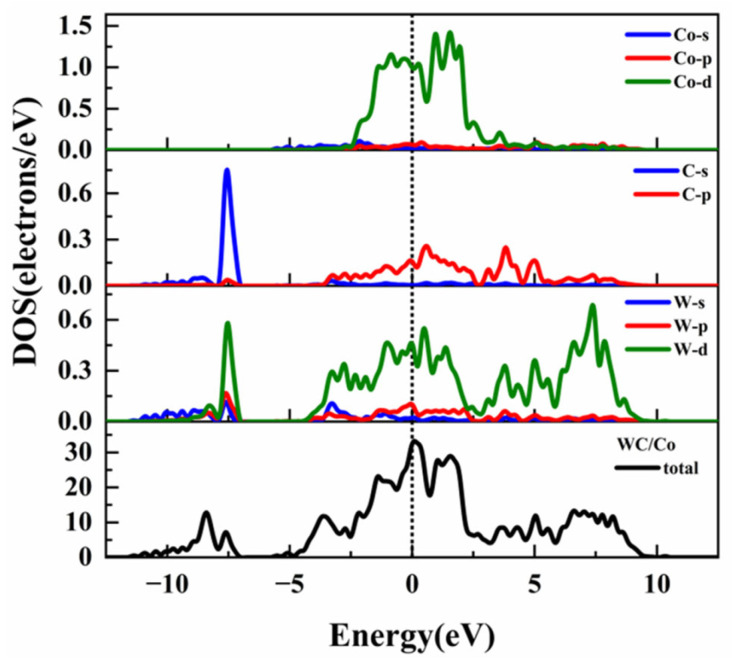
WC/Co density of states and partial density of states.

**Figure 5:**
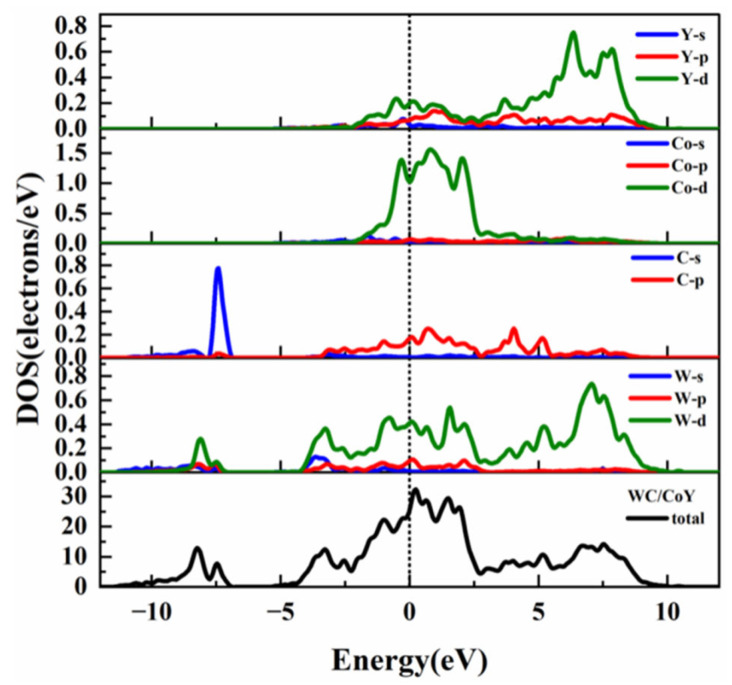
WC/CoY density of states and partial density of states.

To gain deeper insights into the influence of the Y element on the electronic structure and bonding properties of the WC/Co interface, we calculated and compared the total density of states (TDOS) and partial density of states (PDOS) for both the WC/Co and WC/CoY interfaces. This analysis aims to reveal, at the electronic level, the specifics of orbital hybridization and charge redistribution following the introduction of Y atoms, thereby providing a theoretical foundation for the enhancement of interfacial bonding strength. The DOS describes the distribution of electrons or other quantum states in a material across energy space. The TDOS quantifies the distribution of all electronic states at different energy levels in a material. It is commonly used to classify the material’s conductivity type (metal, semiconductor, or insulator), determine the band gap size, and analyze the overall energy characteristics of the electronic states. The Partial Density of States (PDOS) decomposes the total density of states by atom type, atomic orbital (such as s, p, d, f), or spin channel. This helps reveal the atomic-orbital origins of electronic states within specific energy ranges, analyze chemical bonding properties, magnetic behavior, catalytically active sites, and more. Together, TDOS and PDOS provide the microscopic electronic-structure foundation for understanding macroscopic material properties—such as electrical, optical, magnetic, and chemical behavior—and serve as a key bridge between theoretical calculations and experimental performance. In this work, based on the PBE-GGA method, we calculated and analyzed the TDOS and PDOS of the equilibrium interface configurations of WC/Co and WC/CoY. This comparison allows us to evaluate their bonding strength and performance and to understand their bonding characteristics. The TDOS and PDOS results for WC/Co and WC/CoY are presented in Figure 4 and Figure 5, respectively.

As shown in Figure 4, the TDOS and PDOS diagrams of WC/Co. It can be seen from the PDOS diagram that the energy in the range of −10 eV to −5 eV is mainly contributed by the C-s and W-d orbitals, the energy WC/Co in the range of −5 eV to 5 eV is mainly contributed by the W-d, C-p, and Co-d orbitals, and the energy in the range of 5 eV to 10 eV is mainly contributed by the W-d orbitals. As shown in Figure 5, it is the density of states and partial density of states of WC/CoY. The energy in the range of −10 eV to −5 eV is mainly contributed by the C-s and W-d orbitals, and the energy in the range of −5 eV to 5 eV is mainly contributed jointly by the W-d, C-p, Co-d, and Ce-d orbitals. The energy in the range of 5 eV to 10 eV is mainly contributed by the W-d and Ce-d orbitals together.

From the total density of states (TDOS) plots in Figure 4 and Figure 5, it can be observed that the TDOS profiles of the two models are generally similar. In both cases, no band gap is present at the Fermi level, and the density of states exhibits non-zero values, confirming that WC/Co and WC/CoY both display metallic characteristics [30,39]. At the Fermi level, the TDOS value for WC/Co is 32, which is notably higher than the value of 26 for WC/CoY. This indicates that the metallic character of WC/Co is stronger, while that of WC/CoY is comparatively weaker. Furthermore, prominent peaks are observed near the Fermi level in the total density of states of both materials, suggesting that both cemented carbides possess high electrical conductivity. The Fermi-level peak of WC/CoY is sharper than that of WC/Co, which can be attributed to the hybridization among Y-d, W-d, and Co-d orbitals, resulting in a hybridized resonance peak [44].

From the partial density of states (PDOS) plots in Figure 4 and Figure 5, a significant resonance peak between the Y-p orbital and the W-d orbital can be identified, indicating hybridization and the formation of covalent bonds between Y and W atoms. Compared to the W-Co bonding at the WC/Co interface, the Y–W hybridization enhances the covalent character of the bonds. As a result, WC/CoY cemented carbide exhibits higher hardness than WC/Co. This analysis provides a theoretical basis for the experimentally observed enhancement in hardness and wear resistance imparted by the addition of Y in cemented carbides. In summary, the density of states analysis indicates that Y doping induces significant orbital hybridization near the Fermi level (particularly between Y-p and W-d orbitals), leading to the formation of stronger covalent bond character. This directly explains, from an electronic structure perspective, the fundamental reason behind the enhanced interfacial bonding and the improved hardness and wear resistance of the material after Y doping.

### 3.3. Charge Density

To further visualize the changes in chemical bonding nature at the interface induced by Y doping, we plotted the electron localization function (ELF) maps for both the WC/Co and WC/CoY interfaces. ELF analysis provides an intuitive representation of the degree of electron localization in real space, allowing clear differentiation among the characteristics of covalent, metallic, and ionic bonds. This is crucial for corroborating the formation of covalent bonds inferred from the earlier density of states analysis.

**Figure 6:**
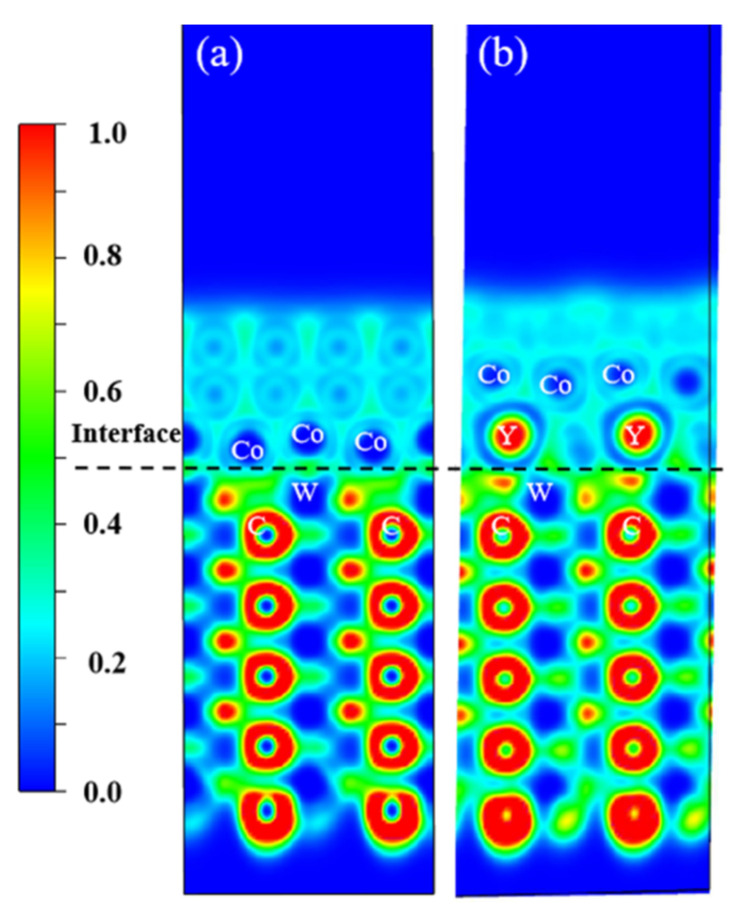
Electron localization function (ELF): (**a**) WC/Co; (**b**) WC/CoY.

Figure 6 presents the Electron Localization Function (ELF) maps for WC/Co and WC/CoY. In these plots, an ELF value of 1 corresponds to regions of covalent bonding or lone-pair electron states, where electrons are fully localized; a value of 0.5 reflects the free-electron-gas behavior characteristic of metallic bonding; and a value of 0 represents a typical ionic-bond region with complete electron delocalization.

Figure 6a displays the ELF map for WC/Co. The WC region shows high localization, consistent with the strongly covalent W–C bonds typical of carbides. At the interface, moderate localization is observed, indicating the mixed metal-covalent character of the W–Co bonds. In contrast, the upper Co region exhibits the delocalized electron distribution characteristic of metallic bonding. Figure 6b shows the corresponding ELF map for WC/CoY. Here, the interface exhibits a higher degree of electron localization than that in WC/Co, manifesting as a mixture of medium and high ELF values. The presence of strong electron localization at the interface suggests the formation of additional covalent bonds—such as Y–W bonds—alongside the existing Co–W bonds. This enhancement in covalent bonding at the interface with Y addition aligns with the hybrid-resonance peak between Y-p and W-d orbitals observed in the PDOS (Figure 5). The introduction of Y significantly increases electron localization at the interface, with the most localized areas corresponding to Y–W atomic positions, confirming the formation of strong covalent bonds through orbital hybridization. These stronger covalent interactions directly contribute to the improved hardness and wear resistance of the alloy. Moreover, the continuous electron distribution across the interface and the absence of weakly bonded regions further enhance the structural stability of the material.

The ELF analysis visually confirms that the introduction of Y leads to higher electron localization in the WC/CoY interface region, clearly indicating the formation of strong Y–W covalent bonds. This aligns with and corroborates the conclusions drawn from the PDOS analysis. Together, they provide evidence from the perspective of charge distribution for the atomic mechanism through which Y strengthens interfacial bonding by enhancing covalent character.

### 3.4. Microstructure Morphology

To verify the theoretical predictions and investigate the influence of Y on the alloy microstructure during the actual sintering process, we examined the microstructures of cemented carbides with different Y contents. The focus lies on analyzing how Y regulates the WC grain size, morphology, and the distribution of the Co binder phase, thereby establishing the link between the microstructure and the macroscopic properties.

**Figure 7:**
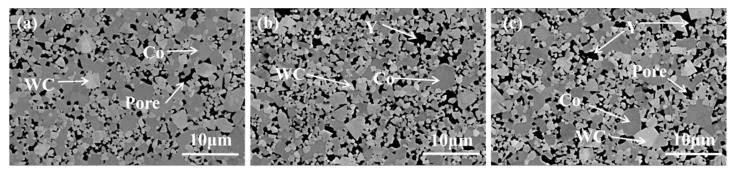
SEM images of the alloy: (**a**) Y-0 wt.%; (**b**) Y-0.5 wt.%; (**c**) Y-1 wt.%.

Figure 7 shows the microstructural morphology of WC–Co-based cemented carbides with different Y contents. The measurement results obtained by Image Pro Plus 6.0 software show that the average grain size of WC in WC-Co is 1.9 μm, while the average grain size of WC with the addition of 0.5 wt.% Y is 1.4 μm, and the average grain size of WC with the addition of 0.5 wt.% Y is 1.5 μm. As shown in Figure 7a, the microstructure of the pure WC–Co cemented carbide reveals that the Co binder phase surrounds and bonds the WC particles together. The Co phase is clustered in patches, and the overall grain distribution is non-uniform, with abnormal grain growth resulting in polygonal WC grains of relatively large size. Figure 7b presents the microstructure after the addition of 0.5 wt.% Y. It can be clearly observed that the introduction of Y inhibits grain growth, leading to a refined and homogeneous grain structure, with the WC grains being the finest among the studied conditions. The refined and homogeneous grain structure observed in Y-doped samples can be attributed to the lower interfacial energy predicted by first-principles calculations, which enhances interfacial stability and inhibits abnormal grain growth. The uniform dispersion of the Co phase in Y-containing alloys aligns with the enhanced interfacial bonding predicted by charge density analysis, promoting better WC–Co cohesion. Moreover, unlike the clustered Co distribution in Figure 7a, the Co phase in Figure 7b is uniformly dispersed, effectively bonding the WC particles. Figure 7c displays the microstructure of the cemented carbide with 1.0 wt.% Y added. The grain structure remains relatively fine and uniform, representing an improvement over the pure WC–Co material. However, compared with the 0.5 wt.% Y condition, some larger WC grains begin to appear in this alloy.

**Figure 8:**
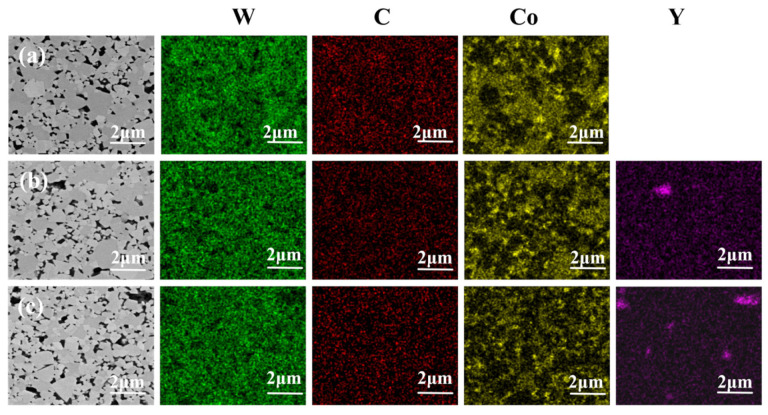
EDS of alloys: (**a**) Y-0 wt.%; (**b**) Y-0.5 wt.%; (**c**) Y-1 wt.%.

Figure 8 presents the corresponding EDS results for the WC-based cemented carbides with different Y contents. Combined with the micrographs in Figure 7, it is evident that in the rare earth-free WC–Co carbide, the Co distribution is uneven and aggregated into patches. With the addition of Y, the distribution of the Co phase becomes more uniform throughout the alloy. At 0.5 wt.% Y, the Y itself is distributed homogeneously, whereas at 1.0 wt.% Y, a slight aggregation of Y is observed. These results indicate that the addition of Y is beneficial only up to an optimal amount, beyond which uniformity may be compromised.

### 3.5. Mechanical Properties

Based on the aforementioned theoretical predictions and microstructural observations, this section systematically tests the macro-mechanical properties of the materials, including density, hardness, and fracture toughness. The aim is to quantitatively evaluate the actual improvement effect of Y doping on the comprehensive mechanical performance of the cemented carbide, and to thereby link the macroscopic properties with the underlying interfacial mechanisms and microstructural evolution.

**Figure 9:**
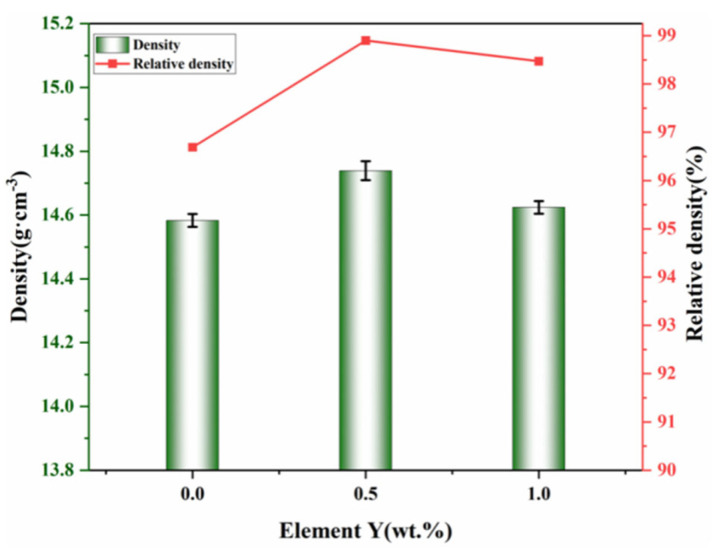
Density and relative density.

Figure 9 presents the density and relative density values of the three types of cemented carbides. As can be seen, the addition of 0.5 wt.% Y increases both the density and relative density of the material. In contrast, at a higher Y content of 1 wt.%, a slight decrease in both density and relative density is observed. This trend can be explained by the dual role of Y during sintering. When added in an appropriate amount (0.5 wt.%), the Y solute enhances the fluidity and wettability of the Co binder during liquid-phase sintering. This promotes a more uniform distribution of Co and WC, inhibits excessive grain growth, refines the microstructure, and reduces the formation of closed pores, thereby aiding densification. However, when the Y content is increased to 1 wt.%, the EDS mapping in Figure 8 shows signs of Y aggregation. Such clustering of rare earth elements tends to create defects that act as barriers to full densification, ultimately leading to a slight decline in both density and relative density. These results demonstrate that while a moderate addition of Y improves microstructural homogeneity and densification, excessive Y can induce inhomogeneity and compromise the consolidation of the alloy.

**Figure 10:**
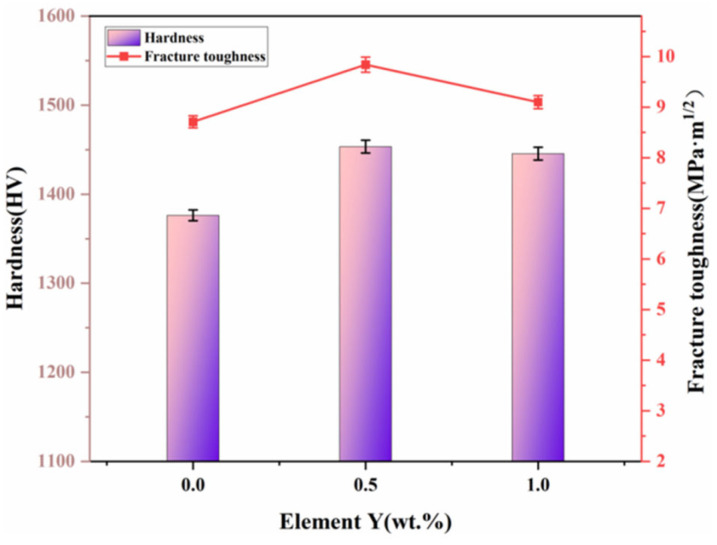
Vickers hardness and fracture toughness.

Figure 10 displays the Vickers hardness and fracture toughness of the cemented carbides with varying Y contents. The calculation formula is as follows [45]:

(4) $$K_{IC}=0.15\sqrt{\frac{HV_{30}}{\Sigma L}}$$

Here, *HV*_30_ is the Vickers hardness obtained under a 30 kg load and Σ*L* is the total length of the crack.

The alloy without Y exhibits the lowest values, with a Vickers hardness of (1376 ± 1.2) HV and a fracture toughness of (8.71 ± 0.12) MPa·m^1/2^. In contrast, the alloy with 0.5 wt.% Y achieves the highest performance, showing a hardness of (1454 ± 1.3) HV and a fracture toughness of (9.84 ± 0.15) MPa·m^1/2^. When the Y content is increased to 1 wt.%, the corresponding values are (1445 ± 1.2) HV and (9.10 ± 0.13) MPa·m^1/2^.

From a first-principles perspective, WC/CoY presents a smaller interfacial area compared to WC/Co. After the removal of the vacuum layer, the unit-cell volume decreases, leading to lower interfacial energy and enhanced interfacial stability. These changes contribute to a more uniform increase in the density and relative density of the resulting cemented carbide. At the interface, Y and W atoms hybridize and form strong covalent bonds, thereby strengthening the interfacial bonding. In metal matrix composites, such robust interfaces act as barriers to dislocation motion, improving the material’s resistance to plastic deformation. Consequently, the strengthened interfaces in WC/CoY indirectly contribute to the elevated hardness observed in these cemented carbides.

### 3.6. Friction and Wear Performance

Wear resistance is a critical service performance of cemented carbide. This section, through friction and wear experiments, aims to investigate the effects of Y addition on the alloy’s friction coefficient, wear rate, and wear morphology, and to explain the synergistic mechanisms behind the enhanced wear performance from multiple perspectives, such as interfacial strengthening and microstructural refinement.

**Figure 11:**
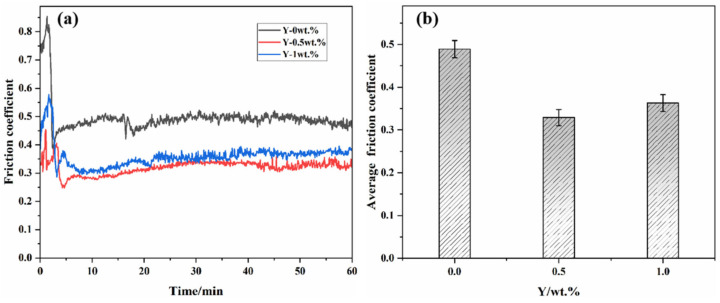
(**a**) Coefficient of friction. (**b**) Average coefficient of friction.

Figure 11 presents the friction coefficients and average friction coefficients of the three types of cemented carbides during a one-hour reciprocating friction test. As shown in Figure 11a, the variation trends of the coefficient of friction (COF) curves for the different samples are generally similar. In the initial 5-minute running-in period, the COF gradually increases, after which the curve stabilizes, indicating the onset of a steady-state friction stage [46,47,48]. The average COF of the Y-free cemented carbide is the highest (0.489), while that of the alloy with 0.5 wt.% Y is the lowest (0.329). When the Y content increases to 1 wt.%, the average COF rises slightly to 0.363. These results demonstrate that the addition of Y can reduce the friction coefficient of the hard alloy; however, the benefit does not increase monotonically with Y content—the performance at 1 wt.% Y is inferior to that at 0.5 wt.%.

Based on first-principles calculations, the introduction of Y increases the relative density of the cemented carbide and stabilizes the interfacial bonding. The hybridization between Y and W atoms forms strong covalent bonds across the interface, which enhances the interfacial bond energy and consequently raises the hardness of the material. The alloy with 0.5 wt.% Y exhibits improved relative density, hardness, and toughness. Under the same reciprocating friction and wear stress, it is less prone to generating wear debris and therefore shows higher wear resistance. In contrast, the alloy with 1 wt.% Y experiences a decline in wear resistance due to excessive rare earth addition, which leads to clustering of Y and the associated microstructural inhomogeneity.

**Figure 12:**
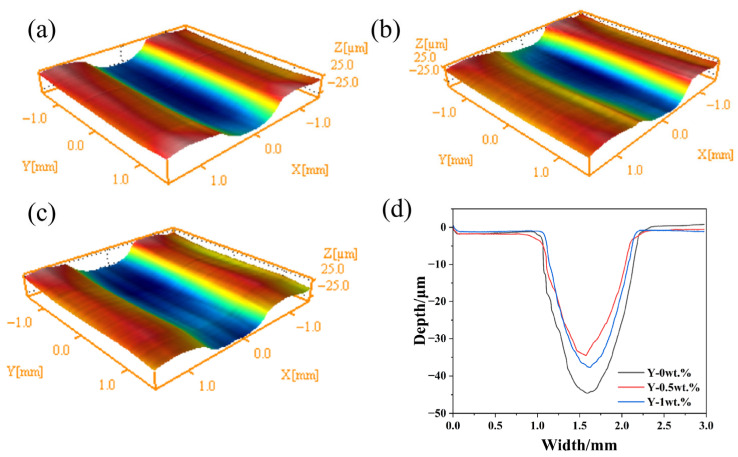
Three-dimensional contour of wear trace: (**a**) Y-0 wt.%; (**b**) Y-0.5 wt.%; (**c**) Y-1 wt.%; (**d**) 2D outline of the wear marks.

Figure 12 presents the 2D and 3D surface profiles obtained after the friction and wear tests. As shown, the wear tracks exhibit a typical “U”-shaped geometry with smooth inner walls and no prominent protrusions, indicating that the wear mechanisms are essentially similar across all the alloys. Detailed measurements from the one-hour reciprocating friction tests under an 80 N load are summarized in Table 2. For the Y-free cemented carbide, the wear depth is the greatest (44.001 μm), with a cross-sectional area of 0.0350 mm^2^ and a wear volume of 0.1747 mm^3^. With the addition of 0.5 wt.% Y, the wear track becomes shallowest (33.093 μm), and both the cross-sectional area (0.0229 mm^2^) and wear volume (0.1144 mm^3^) decrease significantly. When the Y content is increased to 1 wt.%, however, the wear depth increases again to 36.641 μm, accompanied by a cross-sectional area of 0.0257 mm^2^ and a wear volume of 0.1285 mm^3^. These results demonstrate that an appropriate addition of Y effectively enhances the wear resistance of the cemented carbide, while an excessive amount leads to a decline in performance.

**Figure 13:**
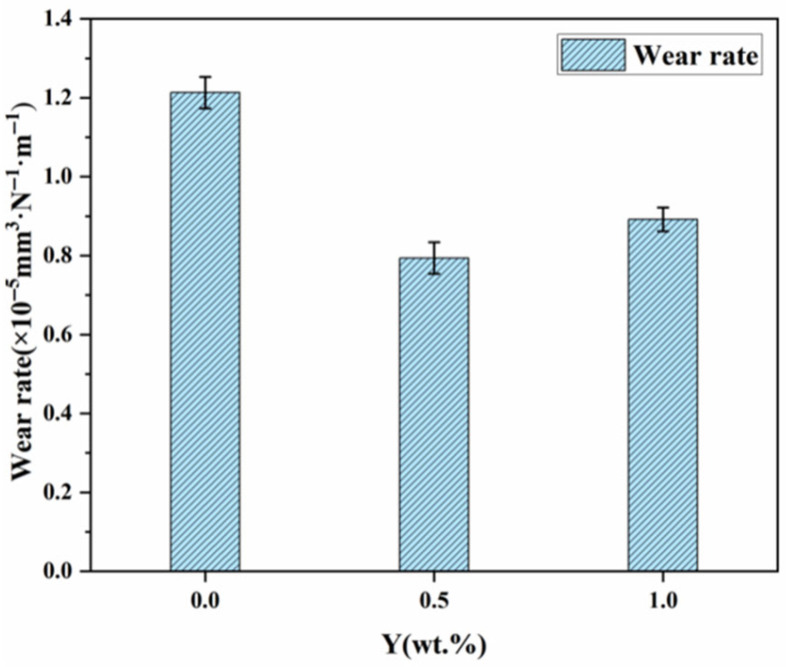
The wear rate of the alloy.

For a more direct comparison of wear behavior, Figure 13 plots the wear rates of the three materials after the reciprocating tests. The Y-free alloy exhibits the highest wear rate (1.213 × 10^−5^ mm^3^·N−^1^·m−^1^). The lowest wear rate is observed for the alloy with 0.5 wt.% Y (0.794 × 10−^5^ mm^3^·N−^1^·m−^1^), whereas increasing the Y content to 1 wt.% raises the wear rate to 0.892 × 10−^5^ mm^3^·N−^1^·m−^1^. Thus, an excess of Y is not beneficial for improving the wear resistance of the cemented carbide. The reduced wear rate and friction coefficient in Y-doped alloys are directly linked to the enhanced interfacial hardness and cohesion predicted by first-principles results. The formation of a stable oxide layer during wear may also be facilitated by the active electronic states of Y, as suggested by the density of states analysis. The decline in wear resistance at 1.0 wt.% Y reflects the theoretical limit of beneficial Y incorporation, beyond which interfacial defects and inhomogeneity prevail.

**Figure 14:**
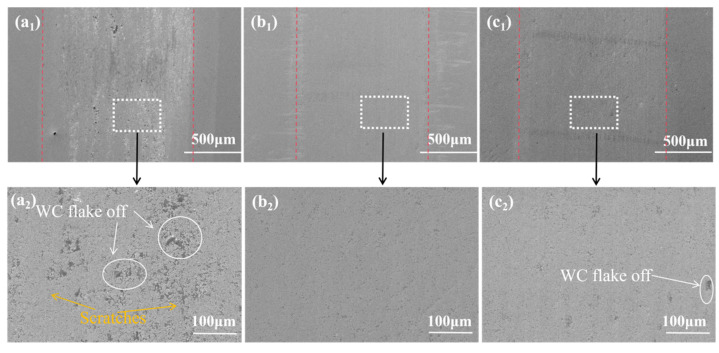
Alloy surface grinding marks of the SEM: (**a_1_**,**a_2_**) Y-0 wt.%; (**b_1_**,**b_2_**) Y-0.5 wt.%; (**c_1_**,**c_2_**) Y-1 wt.%.

Figure 14 displays the wear scar morphologies after reciprocating friction tests conducted under identical load and duration. The alloy without Y addition (0 wt.% Y) exhibits severe wear, with numerous scratches visible across the wear surface. In certain regions, severe fragmentation and spalling of WC particles have led to the formation of cracks and pits, and the wear tracks are comparatively wide. With the introduction of Y, the wear tracks become markedly smoother, and cracks and pits are nearly eliminated. Among the Y-containing alloys, the material with 0.5 wt.% Y shows the best wear scar morphology. Although a few pits appear on the wear tracks of the alloy with 1 wt.% Y, its overall morphology remains significantly better than that of the Y-free alloy.

**Figure 15:**
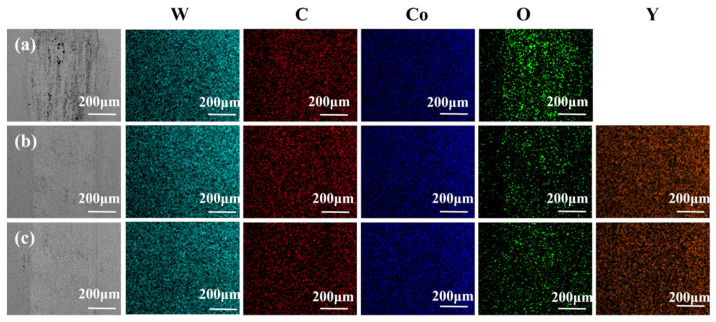
Alloy surface grinding marks of the EDS: (**a**) Y-0 wt.%; (**b**) Y-0.5 wt.%; (**c**) Y-1 wt.%.

Figure 15 presents the corresponding EDS elemental mapping of the wear scars. The W, C, Co, and Y elements are uniformly distributed, while O is enriched within the wear scar regions. In all three cemented carbides, O enrichment is observed at the wear tracks, and its extent correlates positively with the wear severity. The most severely worn 0 wt.% Y alloy shows the most pronounced O enrichment, whereas the least worn 0.5 wt.% Y alloy exhibits the lowest O concentration. For the 1 wt.% Y alloy, with intermediate wear, O begins to display a locally concentrated distribution. This oxygen enrichment results from high-temperature oxidation at the contact interface during reciprocating friction.

Furthermore, the Y-free WC–Co cemented carbide possesses relatively low density and weak intergranular cohesion, making it prone to particle pull-out during friction, which accelerates wear. Its insufficient hardness also leads to deep scratches and localized spalling. With the appropriate addition of Y, the overall properties of the cemented carbide—particularly hardness and relative density—are improved, intergranular cohesion is strengthened, and deformation and cracking are effectively suppressed. Consequently, the 0.5 wt.% Y alloy demonstrates the mildest oxidation and the best wear resistance.

The core mechanism by which the rare earth element Y enhances the properties of WC/Co cemented carbide lies in a series of synergistic microstructural optimizations triggered by the highly active yttrium atoms released during sintering: First, yttrium atoms preferentially segregate at phase boundaries, where they capture impurities such as oxygen and sulfur, resulting in grain boundary purification and strengthening, which significantly improves interfacial bonding strength [49]. Second, yttrium atoms pin the surfaces of WC grains, effectively suppressing abnormal grain growth and yielding a fine-grained structure that contributes to Hall–Petch strengthening. Simultaneously, a portion of yttrium dissolves into the Co binder phase, enhancing its strength and improving its wettability and distribution on WC grains. These structural modifications collectively contribute to the strengthening and toughening of the alloy—fine-grain strengthening and solid solution strengthening directly increase hardness and strength, while the strengthened interfaces alter crack propagation paths from brittle intergranular fracture to more energy-consuming transgranular fracture or complex deflection. This results in a significant improvement in fracture toughness alongside increased hardness. During friction and wear processes, the activity of yttrium further promotes the formation of a dense and strongly adherent yttrium-containing composite oxide film on the surface. This film acts as an effective lubricant and diffusion barrier, reducing adhesive and diffusional wear. Therefore, the synergistic enhancement of hardness, toughness, and surface tribochemical characteristics ultimately leads to a comprehensive improvement in the alloy’s resistance to abrasive wear, chipping, and spalling under severe service conditions.

### 3.7. Limitations and Prospects of Theoretical Calculations

The first-principles calculations in this study provide key insights into the atomic-scale behavior of yttrium (Y) at the WC/Co interface, but it is essential to clearly emphasize the simplified nature of the computational model and the differences from the actual microstructure of polycrystalline materials. Our simulations are based on an ideal, coherent WC(0001)/Co(111) interface model with a specific orientation, with Y atoms placed at a predetermined low-energy site. This idealized setup helps reveal fundamental electronic structures and bonding mechanisms at the interface; however, the actual microstructure of WC–Co cemented carbides is far more complex. First, real materials involve various combinations of WC crystal planes and Co binder phase interfaces, as well as incoherent interfaces containing defects such as steps and vacancies. These different interface structures may exhibit significantly different Y segregation energies and binding strengths. Second, the finite supercell size and periodic boundary conditions may introduce artificial lattice strain and limit the investigation of long-range strain fields and larger-scale interface defects, such as dislocations.

Despite these simplifications, the computational results of this study show a high degree of consistency with experimental observations. The theory predicts interface strengthening and the formation of Y–W covalent bonds, which align with the experimentally observed simultaneous improvements in hardness, toughness, and wear resistance. This indicates that the constructed ideal interface model successfully captures the most essential physical mechanism of Y doping, namely, strengthening the interface bonding through strong localized electronic interactions. However, the model does not encompass all microstructural complexities, such as interface diversity and the statistical distribution of Y. Future research could adopt larger-scale models, investigate a wider variety of interface configurations, or integrate thermodynamic calculations with machine learning methods to provide a more comprehensive description of the role of Y in complex polycrystalline systems. This would enable more precise quantitative prediction and design of material properties.

## 4. Conclusions

In this study, first-principles calculations were adopted to comparatively analyze the interfacial energy, electronic density of states, and charge density characteristics of WC/Co and WC/CoY structures. To test the computational reliability, WC-10Co, WC-10Co-0.5Y, and WC-10Co-1Y alloys were prepared by the activated liquid phase sintering process, and their microstructure, mechanical properties, and friction and wear characteristics were systematically characterized. Based on the comprehensive calculation of simulation and experimental data, the following conclusions are drawn:

1. By calculating the surface energy and interface energy, it was found that both interface structures have mechanical stability. The structural interface can be reduced after adding the Y element, and the interface combination is stronger. When the surface energy increases, the toughness of the material improves.
2. By calculating the electronic density of states and charge density of the WC/Co and WC/CoY models, it was found that hybridization occurred around the Y atoms, forming new chemical bonds. Analyzing the local charge density, it can be known that strong covalent bonds were formed at the WC/CoY interface, making the interface bonding more stable, increasing the hardness and wear resistance of the cemented carbide.
3. Three kinds of cemented carbides, namely WC-Co, WC-10Co-0.5Y, and WC-10Co-1Y, were prepared by activated liquid phase sintering. By observing the microstructure morphology and conducting mechanical tests and friction and wear tests, it was concluded that the cemented carbide with Y-0.5 wt.% added has the best microstructure morphology, with the best hardness and fracture toughness, reaching (1454 ± 1.3) HV and (9.84 ± 0.15) MPa·m^1/2^, and a wearrate of 0.794 × 10^−5^ mm^3^·N^−1^·m^−1^. It is more wear-resistant under the same conditions, which is consistent with the first-principles calculation results.

## Figures and Tables

**Table 1:** The calculated values for the WC/Co and WC/CoY interfaces.

Parameters	E_interface_ (eV)	E_WC_ (eV)	E_binder_ (eV)	A (Å^2^)	W_ab_ (J/m^2^)	γ (J/m^2^)
WC/Co	−407.432	−324.713	−78.238	22.164	3.283	3.807
WC/CoY	−407.356	−324.7127	−79.240	21.808	3.355	3.773

**Table 2:** Parameters related to friction and wear.

Sample	Depth (μm)	Width (mm)	Cross-Sectional Area (mm^2^)	Volume Loss (mm^3^)	Sample
Y-0 wt.%	44.001	1.371	0.0350	0.1747	Y-0 wt.%
Y-0.5 wt.%	33.093	1.375	0.0229	0.1144	Y-0.5 wt.%
Y-1 wt.%	36.641	1.167	0.0257	0.1285	Y-1 wt.%

## Data Availability

The original contributions presented in this study are included in the article. Further inquiries can be directed to the corresponding author.
